# Supplementary material for: Specificity of cranial cutaneous manipulations in modulating autonomic nervous system responses and physiological oscillations: A controlled study
Source: PLoS One. 2025 Feb 27;20(2):e0317300. doi: 10.1371/journal.pone.0317300 (PMC11867383; doi:10.1371/journal.pone.0317300)
Supplement: Attachment — Submitted filename: Response_to_reviewers.docx [file pone.0317300.s001.docx]

Dear Dr. Sanaullah Sajid,

thank you very much for the quick and appreciative revision of our manuscript. We have improved the manuscript according to the reviewer’s and your suggestions and requests. Please find our comments and changes as response below.

With kind regards on behalf of the authors,

Micha Keller

PONE-D-24-41518

Specificity of Cranial Cutaneous Manipulations in Modulating Autonomic Nervous System Responses and Physiological Oscillations: A Controlled Study

PLOS ONE

Dear Dr. Keller,

Thank you for submitting your manuscript to PLOS ONE. After careful consideration, we feel that it has merit but does not fully meet PLOS ONE’s publication criteria as it currently stands. Therefore, we invite you to submit a revised version of the manuscript that addresses the points raised during the review process.

Please submit your revised manuscript by Jan 15 2025 11:59PM. If you will need more time than this to complete your revisions, please reply to this message or contact the journal office at <a href="mailto:plosone@plos.org">plosone@plos.org. When you're ready to submit your revision, log on to https://www.editorialmanager.com/pone/ and select the 'Submissions Needing Revision' folder to locate your manuscript file.

Please include the following items when submitting your revised manuscript:

- A rebuttal letter that responds to each point raised by the academic editor and reviewer(s). You should upload this letter as a separate file labeled 'Response to Reviewers'.
- A marked-up copy of your manuscript that highlights changes made to the original version. You should upload this as a separate file labeled 'Revised Manuscript with Track Changes'.
- An unmarked version of your revised paper without tracked changes. You should upload this as a separate file labeled 'Manuscript'.

If you would like to make changes to your financial disclosure, please include your updated statement in your cover letter. Guidelines for resubmitting your figure files are available below the reviewer comments at the end of this letter.

If applicable, we recommend that you deposit your laboratory protocols in protocols.io to enhance the reproducibility of your results. Protocols.io assigns your protocol its own identifier (DOI) so that it can be cited independently in the future. For instructions see: <https://journals.plos.org/plosone/s/submission-guidelines#loc-laboratory-protocols>. Additionally, PLOS ONE offers an option for publishing peer-reviewed Lab Protocol articles, which describe protocols hosted on protocols.io. Read more information on sharing protocols at <https://plos.org/protocols?utm_medium=editorial-email&utm_source=authorletters&utm_campaign=protocols>.

We look forward to receiving your revised manuscript.

Kind regards,

Sanaullah Sajid, M.Phil/PhD
Academic Editor
PLOS ONE

Journal Requirements:

1. When submitting your revision, we need you to address these additional requirements.

   Please ensure that your manuscript meets PLOS ONE's style requirements, including those for file naming. The PLOS ONE style templates can be found at
   <https://journals.plos.org/plosone/s/file?id=wjVg/PLOSOne_formatting_sample_main_body.pdf> and
   <https://journals.plos.org/plosone/s/file?id=ba62/PLOSOne_formatting_sample_title_authors_affiliations.pdf>

   2. Thank you for stating the following financial disclosure: [HEAD Genuit Stiftung (Aachen, Herzogenrath), Grant/Award Number: HGS03S18072016].

   Please state what role the funders took in the study. If the funders had no role, please state: "The funders had no role in study design, data collection and analysis, decision to publish, or preparation of the manuscript."

   If this statement is not correct you must amend it as needed.

   Please include this amended Role of Funder statement in your cover letter; we will change the online submission form on your behalf.

Reply: As the HGS did not have a role in the study design etc. we have included the statement mentioned above in the updated manuscript.

3. In the online submission form, you indicated that [The data that support the findings of this study are available from the corresponding author, MK, upon reasonable request].

All PLOS journals now require all data underlying the findings described in their manuscript to be freely available to other researchers, either 1. In a public repository, 2. Within the manuscript itself, or 3. Uploaded as supplementary information.

This policy applies to all data except where public deposition would breach compliance with the protocol approved by your research ethics board. If your data cannot be made publicly available for ethical or legal reasons (e.g., public availability would compromise patient privacy), please explain your reasons on resubmission and your exemption request will be escalated for approval.

Reply: Data used for the current publication will still be used for additional analyses and subsequent publications using different methods such as synchronization etc. As soon as this process is finalized we will readily give access to raw data to qualified individuals and institutions.

4. Please review your reference list to ensure that it is complete and correct. If you have cited papers that have been retracted, please include the rationale for doing so in the manuscript text, or remove these references and replace them with relevant current references. Any changes to the reference list should be mentioned in the rebuttal letter that accompanies your revised manuscript. If you need to cite a retracted article, indicate the article’s retracted status in the References list and also include a citation and full reference for the retraction notice.

Reply: As some of the references have been removed and new references have been added as requested by the reviewer, we have also rechecked and updated the reference list accordingly.

[Note: HTML markup is below. Please do not edit.]

Reviewers' comments:

Reviewer's Responses to Questions

**Comments to the Author**

1. Is the manuscript technically sound, and do the data support the conclusions?

The manuscript must describe a technically sound piece of scientific research with data that supports the conclusions. Experiments must have been conducted rigorously, with appropriate controls, replication, and sample sizes. The conclusions must be drawn appropriately based on the data presented.

Reviewer #1: Yes

2. Has the statistical analysis been performed appropriately and rigorously?

Reviewer #1: Yes

3. Have the authors made all data underlying the findings in their manuscript fully available?

The [PLOS Data policy](http://www.plosone.org/static/policies.action#sharing) requires authors to make all data underlying the findings described in their manuscript fully available without restriction, with rare exception (please refer to the Data Availability Statement in the manuscript PDF file). The data should be provided as part of the manuscript or its supporting information, or deposited to a public repository. For example, in addition to summary statistics, the data points behind means, medians and variance measures should be available. If there are restrictions on publicly sharing data—e.g. participant privacy or use of data from a third party—those must be specified.

Reviewer #1: No

4. Is the manuscript presented in an intelligible fashion and written in standard English?

PLOS ONE does not copyedit accepted manuscripts, so the language in submitted articles must be clear, correct, and unambiguous. Any typographical or grammatical errors should be corrected at revision, so please note any specific errors here.

Reviewer #1: Yes

5. Review Comments to the Author

Please use the space provided to explain your answers to the questions above. You may also include additional comments for the author, including concerns about dual publication, research ethics, or publication ethics. (Please upload your review as an attachment if it exceeds 20,000 characters)

Reviewer #1: Firstly, I would like to express my gratitude for your beautiful and innovative research. My comments are as follows:

Reply: We appreciate your valuation of our work.

1-despite all examiners having sufficient experience, doesn't the significant difference in the processes conducted by Examiner 1 compared to other examiners cast doubt on the accuracy of the obtained data?

Reply: We emphasize that, as stated in the manuscript, all examiners underwent rigorous training to ensure consistency in measurement techniques. Our team of osteopathic professionals maintains ongoing dialogue to standardize intervention processes, including Cranial Vault Hold (CVH) and other techniques.

We acknowledge that a majority of measurements were conducted by a single examiner, which could potentially introduce bias. To mitigate this:

1) We employed a linear mixed effects model with a random intercept for examiners to account for baseline variability between examiners.
2) We conducted parallel analyses excluding data from the three additional examiners, yielding similar results.
3) Based on these findings, we determined that using the complete dataset for our final analysis was appropriate and would provide the most comprehensive insights.

For future studies, we recognize the value of having more equally sized groups of examiners. This approach would further strengthen the robustness of the data and minimize potential biases associated with examiner variability.

2- There are 13 articles cited as references in the manuscript that date back to before 2005. It would be better, if possible, to replace them with more recent references.

Reply: We have removed all references dating back to before 2005 which are not absolutely indispensable for a sound understanding of the manuscript (e.g., Nelson 2001, Lambertz 1998, Langhorst, 1984, Ziege 1992). Please check the manuscript for exact changes.

3-The keywords used should be reviewed for compliance with MeSH (Medical Subject Headings) standards, and if possible, fewer keywords should be used.

Reply: We have reduced the number of keywords and have tried to adapt those remaining to standards in compliance with MeSH. However, some of our keywords which we deem important are not listed on MeSH and we have decided to keep them.

4-Considering that a large amount of data has been presented in the article, and one of the important aspects of publishing an article is ensuring it is easily understandable for readers, it is recommended, at the discretion of the esteemed editor, to include a graphical abstract that presents the process and overall results of the work in the article

Reply: We are grateful for this valuable suggestion and have included a graphical abstract for a faster overview on the plethora of data and for visibility.

5-If possible, please use a different chart format to present the data in Table 3. The data dispersion has led to significant fluctuations in the table, and the current format does not effectively convey the results.

Reply: We have checked all tables and changed chart format in Table 3.

6. PLOS authors have the option to publish the peer review history of their article ([what does this mean?](https://journals.plos.org/plosone/s/editorial-and-peer-review-process#loc-peer-review-history)). If published, this will include your full peer review and any attached files.

If you choose “no”, your identity will remain anonymous but your review may still be made public.

**Do you want your identity to be public for this peer review?** For information about this choice, including consent withdrawal, please see our [Privacy Policy](https://www.plos.org/privacy-policy).

Reviewer #1: No
